# Tuberculosis Prevention in South Africa

**DOI:** 10.1371/journal.pone.0122514

**Published:** 2015-04-07

**Authors:** Gwenan M. Knight, Peter J. Dodd, Alison D. Grant, Katherine L. Fielding, Gavin J. Churchyard, Richard G. White

**Affiliations:** 1 TB Modelling Group, Centre for the Mathematical Modelling of Infectious Diseases, London School of Hygiene and Tropical Medicine, London, United Kingdom; 2 TB Centre, London School of Hygiene and Tropical Medicine, London, United Kingdom; 3 School of Health and Related Research, University of Sheffield, Sheffield, United Kingdom; 4 Aurum Institute, Johannesburg, South Africa; 5 School of Public Health, University of Witwatersrand, Johannesburg, South Africa; Cambridge University, UNITED KINGDOM

## Abstract

**Background:**

South Africa has one of the highest per capita rates of tuberculosis (TB) incidence in the world. In 2012, the South African government produced a National Strategic Plan (NSP) to control the spread of TB with the ambitious aim of zero new TB infections and deaths by 2032, and a halving of the 2012 rates by 2016.

**Methods:**

We used a transmission model to investigate whether the NSP targets could be reached if immediate scale up of control methods had happened in 2014. We explored the potential impact of four intervention portfolios; 1) “NSP” represents the NSP strategy, 2) “WHO” investigates increasing antiretroviral therapy eligibility, 3) “Novel Strategies” considers new isoniazid preventive therapy strategies and HIV “Universal Test and Treat” and 4) “Optimised” contains the most effective interventions.

**Findings:**

We find that even with this scale-up, the NSP targets are unlikely to be achieved. The portfolio that achieved the greatest impact was “Optimised”, followed closely by “NSP”. The “WHO” and “Novel Strategies” had little impact on TB incidence by 2050. Of the individual interventions explored, the most effective were active case finding and reductions in pre-treatment loss to follow up which would have a large impact on TB burden.

**Conclusion:**

Use of existing control strategies has the potential to have a large impact on TB disease burden in South Africa. However, our results suggest that the South African TB targets are unlikely to be reached without new technologies. Despite this, TB incidence could be dramatically reduced by finding and starting more TB cases on treatment.

## Introduction

South Africa suffers from an extremely high burden of tuberculosis (TB) disease: in 2013 it was one of the top six countries ranked by number of incident TB cases (0·4–0·6 million) [1]. Globally, TB is the second leading cause of death from an infectious disease [1].

Several targets for TB control have been set by the United Nations (UN), the World Health Organization (WHO) and the South African government for a range of indicators, with different degrees of specificity and feasibility. For example, the UN Millennium Development Goal, set in 2000 for 2015, to have a falling TB disease incidence globally,[2] has already been reached [1]. However, in the Africa region, primarily because of the rise of HIV, the WHO STOP TB goal to halve TB prevalence and deaths between 1990 and 2015,[3] is unlikely to be achieved [1]. The South African government have produced their own National Strategic Plan (NSP) for tackling “HIV, STIs and TB” [4]. This plan sets out ambitious aims: to halve TB incidence and TB related mortality between 2012 and 2016, with zero new infections and zero TB mortality by 2032.

To reach the NSP targets, the prevention strategies outlined in the plan are “based on current knowledge” [4]. Other interventions under development, such as new TB vaccines, need to be pursued but cannot be used to reach the 2016 target. Efforts to achieve the NSP targets must therefore be based on existing control technologies. New combinations and optimising existing strategies, such as providing antiretroviral therapy (ART) to all people living with HIV (PLHIV) with TB disease and isoniazid preventive therapy (IPT) to all PLHIV pre-ART, may be needed to fulfil the potential of existing methods.

Our aim was to use a transmission model to make a preliminary investigation as to whether the NSP targets could be reached if immediate scale up of existing control methods had happened at the start of 2014.

## Methods

### Model and basecase scenario

We adapted a previously published,[5] stochastic individual-based simulation model of the transmission dynamics of *M*. *tuberculosis (M*.*tb*.) and fitted the model to current data on TB and HIV epidemiology in South Africa (see S1 File for full details). Briefly, the model includes TB and HIV natural history at an individual-level, with population level incidence sustained by *M*.*tb*. transmission. Latent infection with *M*.*tb*. (which confers partial protection against progression to disease following re-infection) develops into active (sputum smear negative or positive pulmonary) TB disease at a rate that is higher shortly after (re-)infection and varies with age. The model incorporated data on age-structured social mixing patterns, [6] as well as historic and projected trends in demography [7]. Timing of development of TB disease after (re-)infection is influenced by HIV status and CD4 count; HIV status and age influence the probability of TB disease being smear-positive. The model is built using C. All parameters are found in Table A in S1 File.

In terms of the natural history of TB, individuals are born without *M*.*tb* infection; can acquire an *M*.*tb* infection which may, or may not develop into active TB disease; and individuals may, or may not have their active TB disease detected and initiate treatment. The force-of-infection for TB is proportional to the prevalence of active TB, weighted by the infectiousness of each prevalent TB case. We assume age-dependent mixing between different age groups, parameterized with data from a social contact study carried out in South Africa as part of ZAMSTAR [6]. Upon infection with *M*.*tb*, individuals’ time-since-infection is tracked and they are subject to a hazard of developing pulmonary TB disease per unit time. This hazard is higher in the first 2 years following (re-)infection, with an age-dependence following that used in [8]. In subsequent years, the hazard of activation assumes a lower constant value. Individuals with an *M*.*tb* infection have a partial protection against reinfection [9], however, a successful re-infection is treated identically to an initial infection.

Upon activation to pulmonary TB, 65% of cases in 20 year olds were assumed smear positive among those without HIV infection, following the age pattern reported in [8], smear negative TB assumed 23% as infectious as smear positive TB [10,11]. Active TB without treatment results in death (70% for smear-positive disease; 30% for smear-negative disease) or else self-cure over a timescale of 3 years based on [12]. These alternatives are modelled as proportional hazards with a Weibull-distributed time-to-event. Self-cure leaves individuals with an *M*.*tb* infection, and acts like a new infection in terms of activation risks.

Detection and initiation of treatment in those with active TB disease is assumed to occur with a probability taken as the WHO estimate of the case-detection rate [13]. The timing of detection and treatment initiation occurs at a certain fraction of an individual’s time-to-outcome without detection and treatment (i.e. more rapidly progressing TB disease is detected proportionately more rapidly).

For PLHIV, CD4+ T-cell counts of cells/mm^3^ (CD4) and TB risk were explicitly modelled, as was ART initiation and its effect on CD4 count and life expectancy by age. A proportion of PLHIV can be “linked to care” prior to qualifying for ART *i*.*e*. PLHIV who know their HIV status but have a high CD4 count. This group start ART at the national guideline threshold. The CD4 count at ART start for those detected as HIV positive with a CD4 count under the guideline threshold was taken from a distribution determined by data from a large set of ART cohorts [14]. This distribution has a median CD4 value at ART start of 152 cells/mm^3^. HIV incidence for each scenario was taken from a deterministic model of HIV transmission (see S1 File) [15]. The model was calibrated to the situation in South Africa by varying seven key parameters (see S1 File).

The model basecase scenario assumes continued implementation of control methods as used in South Africa up to mid-2013. This includes IPT to PLHIV at 10% coverage and ART to those PLHIV with active TB at 15% coverage by 2014. Coverage was assumed to rise to 80% coverage by 2015 to reflect the high adherence levels to ART guidelines [16]. In 2010, ART guidelines were revised from initiating those with a CD4 count of less than 200 cells/mm^3^ (2004 guideline), to < 350 cells/mm^3^ with coverage to this group reaching 80% by 2012 [16]. The percentage of TB patients that were detected and started onto treatment was 69% [17]. This was independent of HIV status and was composed of both probability of detection and pre-treatment loss to follow-up. Introduction and scale-up of the GeneXpert System (Cepheid, Sunnyvale, CA) was implicitly included in the basecase scenario and we assumed this had no impact on underlying parameters such as the rate at which patients start treatment as reported in the most recent South African data [18,19]. Parameter values are given in the Table A in the S1 File. The mean of 15,000 simulations was used for each outcome for each scenario. This number of simulations was used so that stochastic error was then reduced below that of the precision presented in the results and we could be confident in impact size to within 1%.

### Interventions

Interventions were assumed to have started together on the 1^st^ of January 2014 and to have had short scale up times (1 year) to factor in time for preparations,[20] whilst leaving a maximum amount of time to achieve impact before the beginning of 2016. The interventions under consideration involve three separate control measures: ART, IPT, and improved TB case management. These were grouped into four portfolios of interventions for investigation, which are outlined below and in Table 1 (full details in S1 File). The portfolios were chosen to represent groups of existing interventions that are recommended in different guidelines (NSP and WHO), or not yet in any (Novel Strategies). We then chose the most effective individual interventions and combined these into one portfolio that was predicted to give maximal impact with current tools (Optimised).

**Table 1:** Summary of the interventions included in each portfolio.

	**ART**			**IPT**				**Periodic active case finding**		**Improved TB case management** *
**Portfolio**	**At CD4 < 350**	**At CD4 < 500**	**Universal test and treat**	**6 months to PLHIV**	**36 months to PLHIV**	**Continuous to PLHIV**	**6 months to HIV negatives**	**Coverage**		
								**Low**	**High**	
**Basecase**	X			X						
**NSP**	X				X			X		X
**WHO**		X			X					
**Novel Strategies**			X			X	X			
**Optimised**			X			X	X		X	X

* Decreased pre-treatment loss to follow-up and improved treatment success.

### Portfolios

1. **NSP.** This portfolio built on the basecase scenario but with higher adherence to the control measures and additional interventions as discussed in the National Strategic Plan [4]. IPT coverage was increased to 53% of newly detected PLHIV (as recorded in a recent HIV counselling and testing campaign)[21] and set at 36 months in duration. The additional interventions were: improved TB case management and periodic active case finding. The former assumed increased treatment success (50% reduction in treatment failure) and a decreased pre-treatment loss to follow-up (by 50%). The latter assumed annual screening rounds (not just enhanced case finding [22]) reaching 30% of the population, as seen in recent HIV testing campaigns,[23] and a high sensitivity test, such as GeneXpert. When taking into account the acceptance rate of screening and pre-treatment loss to follow up, this intervention was assumed to start 16% and 12% of previously undetected smear positive/negative TB on treatment, respectively.
2. **WHO.** This portfolio was motivated by the World Health Organization guidelines on IPT duration and ART [24]. The IPT coverage was assumed to be as in the NSP portfolio, but the ART initiation guideline was increased in 2014 to a CD4 count of < 500 cells/mm^3^ with coverage of those eligible reaching 90% by 2020.
3. **Novel Strategies.** This portfolio contained those interventions that are under consideration, but are not yet included in any guidelines. We built on the NSP portfolio by assuming a) ART was given universally (Universal Test and Treat, UTT): annually, 42% of PLHIV who are not on ART were assumed to start ART via this intervention. No additional TB case detection was included in this ART-only version of UTT; b) Continuous IPT was provided to 53% of PLHIV and c) 6 months of IPT was also provided randomly to the HIV negative population with 20% coverage to investigate the potential new use of a currently existing therapy, shown recently to be highly effective [25].
4. **Optimised.** This portfolio combined the most effective interventions at the realistic coverage levels considered in the other portfolios, with an additional high coverage for periodic case finding as this is advocated for in the National Strategic Plan [4]. It assumed the basecase scenario plus a) UTT; b) 6 months IPT to 20% of HIV negatives; c) continuous IPT to 53% of PLHIV on starting ART; d) 50% decrease in pre-treatment loss to follow-up and an increase in treatment success (a 50% decrease in treatment failure), and e) high coverage (60%) periodic active case finding, with a high sensitivity test such as GeneXpert, starting, annually, 32% and 24% of undetected sputum smear positive/negative TB on treatment.

### Sensitivity analysis

We evaluated the robustness of our conclusions by exploring the “NSP” portfolio impact using a sensitivity analysis of key model parameters. Full details are in the S1 File.

## Results

### Basecase

**Fig 1:**
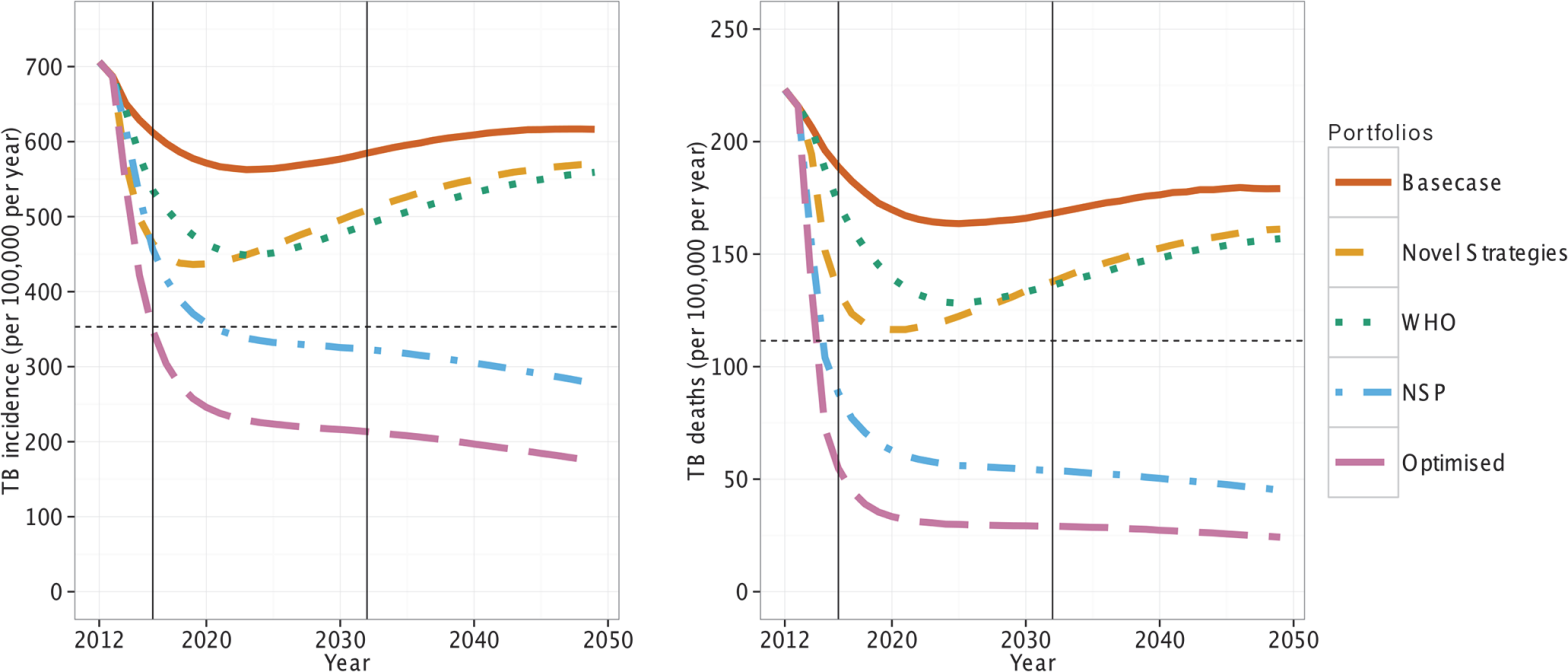
Basecase and impact of the four portfolios on TB incidence and mortality (/100,000/year). The “NSP” and “Optimised” portfolios have the biggest impact. The dashed horizontal lines are the NSP 2016 targets. The solid vertical lines show the timings of the NSP deadlines for 2016 and 2032.

In line with recent data,[26] in the basecase scenario TB incidence and mortality were falling in 2012. The decrease past 2014 was due to declining HIV incidence via maintenance of high ART coverage. Both incidence and mortality flattened from 2020 onwards (Fig 1).

### Intervention Portfolios

Our results suggest, that the “Novel Strategies” and “WHO” portfolios could result in 32% and 22% reductions in TB incidence by 2016 (Fig 1 and Table 2). Both portfolios peak in impact before 2032 and neither achieved greater than 40% reductions in TB incidence or mortality at any time point. The other two portfolios (“NSP” and “Optimised”) had a much larger impact by 2016 (Fig 1 and Table 2). By 2016, the “NSP” portfolio resulted in the prevention of a large number of TB deaths (57%) but only a 32% reduction TB incidence.

**Table 2:** Percentage reduction in TB incidence and mortality in each portfolio compared to 2012 levels.

		2016		2032		2050	
NSP target		50% reduction		Zero new cases and deaths		No NSP target; WHO ‘elimination’ year [3]	
		Incidence	Mortality	Incidence	Mortality	Incidence	Mortality
**Portfolio**	**Basecase**	13	15	18	26	14	21
	**NSP**	32	**57**	55	75	61	80
	**WHO**	22	21	32	40	22	31
	**Novel Strategies**	32	39	29	40	20	29
	**Optimised**	46	**71**	70	86	76	89

Bold, underlined values indicate where the reductions are greater than the NSP targets for 2016 and 2032.

The final, “Optimised”, portfolio nearly achieved the 2016 NSP goals with a 46% and 71% reduction in incidence and mortality respectively (Fig 1 and Table 2). By 2050, both TB incidence and mortality could be reduced by more than 75% using the “Optimised” portfolio. However, TB incidence and mortality remained greater than the required zero new cases and deaths NSP target for 2032.

**Fig 2:**
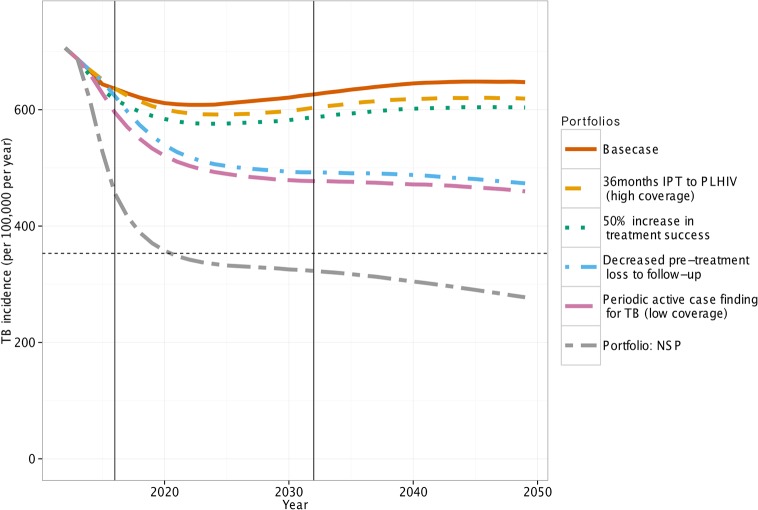
The impact of the intervention components of the “NSP” portfolio on TB incidence (/100,000/year). The dashed horizontal line is the NSP 2016 target. The solid vertical lines show the timing of the NSP deadlines in 2016 and 2032.

When the portfolios were broken down into their contributing interventions, we found that for the high impact portfolios (“NSP” and “Optimised”) most of the impact (on both incidence and mortality) was attributed to reductions in pre-treatment loss to follow up and to periodic (annual) active case finding. In the “NSP” portfolio ~40% of the impact was attributed to the former and ~40% to the latter at low coverage (Fig 2, see S1 File for other portfolio breakdowns).

### Individual Interventions

The only single intervention that reduces both TB incidence and deaths by more than 50% by 2050 was annual active case finding in the general population (Fig 2 and S1 File). By 2032, the high sensitivity, high coverage scenario reduced TB incidence by 48% and the number of TB deaths by 58%. The intervention with the next highest impact was a 50% decrease in the pre-treatment loss to follow up, with a ~30% and 52% reduction in TB incidence and deaths respectively by 2032. Despite the interacting nature of the HIV and TB epidemics, the ART interventions in general have only a modest impact, except for UTT, which had a long-term impact on TB incidence of ~20%. The IPT interventions have little long-term impact on TB incidence or deaths. Providing IPT to HIV negatives gave the biggest impact with a 13% and 20% decrease in TB incidence and deaths respectively by 2050.

### Reaching the targets

The model predicts that even if the “Optimised” portfolio had been immediately implemented in 2014, the South African 2016 aims will not be achieved (Fig 1). The 2032 goals of zero new cases and deaths are similarly unlikely to be achieved, with only two of the portfolios investigated here (“Optimised” and “NSP”) achieving greater than a 50% reduction in incidence and mortality by 2032. Indeed, both these portfolios achieved this reduction before 2016, thus partially fulfilling the NSP 2016 targets. Little difference was seen here in the impact of the portfolios between 2032 and 2050.

Thus, our results suggest that high levels of TB control could be achieved using scale up of current tools, as reductions of over 50% in the burden of TB could be achieved by 2032.

### Sensitivity analysis

Varying key model parameters has little influence on the impact of the primary “NSP” portfolio (S1 File). The parameters chosen were the probability that TB on ART is like that in those without HIV, the protection from re-infection if latently infected, the probability of death if TB is not cleared and the ratio of case detection rates by smear status in those without HIV. The only parameter which generated a greater than 3% difference in the impact of the “NSP” portfolio was the effect of ART on the risk of acquiring TB in PLHIV.

Under all parameter ranges explored, the overall difference in impact of the “NSP” portfolio changed within the range of -6.5% to 1% (see S1 File for more details). This suggests that our conclusion that the “NSP” portfolio would not achieve either of the NSP TB targets is robust.

## Discussion

The results presented here suggest that large decreases in the tuberculosis burden of South Africa could be achieved using existing technologies and simply increasing the numbers of patients starting TB treatment. Despite this, we find that even if these portfolios had been immediately scaled up early in 2014, the 2016 and 2032 NSP targets are unlikely to be achieved. Instead, these targets should be seen as useful for advocacy purposes, and goals to which significant progress can be made using current tools. Of the components explored, active case finding and a reduction in pre-treatment loss to follow up were the most effective and could have a large impact. The individual interventions we considered fell into 3 categories with conclusions that are broadly applicable to other settings: those involving IPT; those involving ART; active case-finding and improved TB case management.

While there is good evidence that IPT is beneficial in reducing TB incidence in individuals with HIV infections,[27] we find that policies involving IPT are likely to have limited population-level impact on TB incidence. This stems from relatively pessimistic assumptions in light of the most recent evidence around the durability of protection from IPT,[28–30] and from the low prevalence of those on IPT in the population. We assumed only 10% of latent infections with *M*. *tb* were cleared by a full course of IPT [28–30] and that a coverage of IPT of only 10% or 53% of those PLHIV who were IPT naïve could be achieved per year. Although targeted at a group of higher risk for TB (those on ART), scenarios with 6 or 36 months of IPT to those starting ART never exceeded a population prevalence of more than 5% on IPT. On the other hand, prescribing IPT to HIV-negative individuals, resulted in an ~15% prevalence in the general population on IPT. This higher population coverage may have resulted in the greater impact of this HIV-negative targeting, but the risks of such an approach due to side–effects [31] would need to be carefully evaluated before it would be considered in practice.

We predict that widespread use of ART should have a noticeable population-level impact on TB incidence as has been predicted previously [5]. In the short-term, this effect is driven by the reduction in individual rates of TB among PLHIV; in the long term, averted HIV infections also make a contribution, especially for UTT. This impact is despite the assumed lack of complementary TB case finding as in standard UTT. Although ART reduces individual rates of TB, those on ART still have 4-7-fold higher rates of TB than the HIV-negative population, and reductions in mortality can result in increased HIV-prevalence. These competing effects are responsible for a plateau in impact in some ART scenarios, and mean that a policy emphasizing ART initiation in those with TB, without bolstering general provision of ART, can increase population TB incidence by keeping those at highest risk of TB alive [32].

While there is a lack of empirical evidence to support the impact of active case-finding in the general population,[33] our modelling suggests that active case-finding and reductions in pre-treatment loss to follow-up, which act by reducing the pool of infectious individuals, are predicted to have a sustained and accumulating reduction on TB incidence. Our active case-finding scenarios, while clearly very intensive, may not be unachievable. They are comparable to levels achieved in previous active case finding interventions where 20% of undiagnosed cases were thought to be diagnosed per 6 monthly campaigns [34]. We did, however, assume that the population is uniformly reachable by case-finding rounds: that extra TB cases initiated on treatment regardless of symptoms would not have higher rates of pre-treatment loss to follow-up, or overload programme capacity, and that TB cases are uniformly infectious over time. Relaxing any of these assumptions would reduce the predicted impact of active case finding. Future work should specifically tackle the random screening assumption, with targeting of high-risk sub-populations. Pairing a form of active case finding with UTT (as is standard for UTT protocols) is likely to be a powerful tool for TB control.

Reductions in pre-treatment loss to follow-up are also predicted to be a potent interventions and are more easily achievable within the current health infrastructure than active case finding, by measures such as increased efficiencies in the diagnostic process, increased education and improved follow-up by healthcare professionals [35]. These measures should enhance the impact of the now widely available GeneXpert. Improvements in treatment initiation levels made important contributions to reducing TB mortality, but resulted in smaller reductions in TB incidence by affecting outcomes after transmission events had already occurred. This large impact of improved case management has been seen in other models of South Africa TB control [36].

Our model does not explicitly include multi-drug resistance TB, however our key conclusions are robust to this limitation. These key conclusions on overall NSP impact were also robust to our sensitivity analysis. However, because of computational resource constraints, this sensitivity analysis was limited in scope. As such, although we believe it is unlikely, we cannot exclude the possibility that there are combinations of baseline parameter values that would lead the model to predict that the NSP scenario could reach the targets. We also designed our interventions with rapid scale-up timings and thus did not tackle the important question of operational feasibility. Our aim instead was to provide estimates of the upper limits of the impact of current technologies. In relation to this, the relatively small difference between the impact of the “Optimised” and “NSP” portfolios suggest that the additional cost of the higher coverage included in “Optimised” would have to be carefully assessed against relative impact: the lower coverage of active case finding and simply improving TB case management as included in the “NSP” could alone be highly successful in the control of TB. As South Africa moves forward with its adoption of the NSP, this work suggests that close attention should be paid to the implementation and coverage of these interventions that were most successful in this model.

### Conclusion

Although current control technologies are unlikely to achieve either of the South Africa targets for TB control, this study predicts that interventions such as active case finding and a reduction in pre-treatment loss to follow up could have a large impact. Our modelling provides evidence on the likely differential relative impact of interventions, needed for future strategy implementation decisions and highlights two important methods that could have a significant impact on future TB burden. We predict that even with the best deployment of the tools we currently have available, new technologies will be needed to reach the 2032 South African TB targets for control; however TB incidence could still be dramatically reduced using only existing control measures.

## Supporting Information

S1 File Supplementary information for “Tuberculosis prevention in South Africa”. (DOCX)
