# Supplementary material for: Tuberculosis Prevention in South Africa
Source: PLoS One. 2015 Apr 7;10(4):e0122514. doi: 10.1371/journal.pone.0122514 (PMC4388715; doi:10.1371/journal.pone.0122514)
Supplement: S1 File — (DOCX) [file pone.0122514.s001.docx]

**Supplementary information for “Tuberculosis prevention in South Africa”**

**Index**

1) Mathematical model details and parameter table (Table A)

2) HIV incidence

3) Details of individual interventions

4) Results of the impact of individual interventions with breakdown plots of each portfolio with component interventions

5) Sensitivity analysis

**1) Mathematical model**

We modified an individual-based simulation model developed for CREATE (the Consortium to Respond Effectively to the TB/AIDS Epidemic)[1-3] to investigate the impact of a shortened first line drug regimen. The model includes age, HIV status and CD4 count, and TB infection history. Timing of development of TB disease after (re-)infection is influenced by HIV status and CD4 count; HIV status and age influence the probability of TB disease being smear-positive. TB infection from TB cases occurs in the community, the latter with age-dependent mixing. The model is built using C. All parameters are found in Table A.

*Natural history of TB*

The TB model structure follows along the lines of the simplified representation in Figure A: individuals are born without *M.tb* infection; can acquire an *M.tb* infection which may, or may not develop into active TB disease; and individuals may, or may not have their active TB disease detected and initiate treatment.

*TB transmission*

The force-of-infection for TB is proportional to the prevalence of active TB, weighted by the infectiousness of each prevalent TB case. We assume age-dependent mixing between different age groups, parameterized with data from a social contact study carried out in South Africa as part of ZAMSTAR [4].

Figure A: Overview model diagram. This diagram shows the schematic progression through TB states in the model, but for simplicity omits mortality and birth, HIV/ART status. The latent, active and treatment states are finer-grained in the model as they track time since entering the state.

*TB in HIV-uninfected individuals*

Upon infection with *M.tb*, individuals’ time-since-infection is tracked and they are subject to a hazard of developing pulmonary TB disease per unit time. This hazard is higher in the first 2 years following (re-)infection, with an age-dependence following that used in [5]. In subsequent years, the hazard of activation assumes a lower constant value. Individuals with an *M.tb* infection have a partial protection against reinfection [6], however, a successful re-infection is treated identically to an initial infection.

Upon activation to pulmonary TB, 65% of cases in 20 year olds were assumed smear positive among those without HIV infection, following the age pattern reported in [5], smear negative TB assumed 23% as infectious as smear positive TB [7,8]. Active TB without treatment results in death (70% for smear-positive disease; 30% for smear-negative disease) or else self-cure over a timescale of 3 years based on [9]. These alternatives are modelled as proportional hazards with a Weibull-distributed time-to-event. Self-cure leaves individuals with an *M.tb* infection, and acts like a new infection in terms of activation risks.

Detection and initiation of treatment in those with active TB disease is assumed to occur with a probability taken as the WHO estimate of the case-detection rate [10]. The timing of detection and treatment initiation occurs at a certain fraction of an individual’s time-to-outcome without detection and treatment (i.e. more rapidly progressing TB disease is detected proportionately more rapidly).

**HIV**

*HIV infection and life-expectancy*

HIV incidence through calendar time was taken from a deterministic model of HIV transmission [11]. HIV infections are distributed by age according to a Weibull distribution with shape parameter k=2.3 and scale s=25.9 years matching the gender-average of data presented in [12]. Life expectancy for those infected by HIV without ART is modelled by a Weibull distribution with parameters (k=2.3, s=13.3 years), from a weighted least-squares fit to the survival data from the CASCADE collaboration [13].

*HIV & TB*

The influence of HIV on the risks of developing TB is mediated through CD4 count, which is modelled continuously for each individual. The CD4 cell count trend and incidence rate ratio for developing TB in HIV-infected individuals not receiving ART are modelled similarly to [14]. Upon infection, CD4 cell count drops by 25% from an initial value of 1000 cells per microliter, followed by a linear decline to zero at death. Individuals’ TB incidence rate ratio (IRR) increases exponentially with decreasing CD4 cell count with a rate 0.36 per 100 cells per microliter. This IRR applies to the hazard of an *M.tb* infection becoming active TB disease as modelled above. HIV-infected individuals are assumed not to have any protection from a prior *M.tb* infection against reinfection disease.

HIV-infected active TB cases were taken to be 45% as likely to be smear positive as HIV-uninfected TB cases [14]. Untreated active TB disease in HIV-infected individuals was assumed to have 100% case-fatality, over a time-scale of 5 months [15], also modelled as a Weibull distribution. The probability of detection and treatment initiation, and its timing, was modelled as for TB in HIV-uninfected individuals.

*ART & ART coverage*

The life expectancy of an individual starting ART was modelled as in [16]: for someone of age *a* initiating ART at CD4 count *CD4_A_* whose CD4 count immediately following seroconversion was *CD4_1_*, their life-expectancy is taken as:

$$max({LE}_{0}-a,0)\times\left( \frac{{CD4}_{A}}{{CD4}_{1}} \right)^{0.5}$$

where *LE_0_* is the individual’s life-expectancy in the absence of HIV infection.

The effect of ART on TB incidence was taken to be equivalent to returning CD4 count to that following seroconversion, resulting in a population IRR of approximately 0.3 for TB in those on ART compared with HIV-infected individuals not on ART.

ART initiation was modelled as for the PopART model used in [17]. Briefly, individuals who were classified as in contact with health services and whose CD4 count was below the threshold of 350 cells per microliter were assumed to commence ART at a rate of 2 per year. The rate of becoming in contact with health services was modelled by a function of the form:

$$A_{max}\times\frac{\left[ \left( t-t_{0} \right)/s \right]^{k}}{1+\left[ \left( t-t_{0} \right)/s \right]^{k}}$$

where *t_0_* was taken as 2004, and the other parameters were fitted to reported South African ART coverage. In this work we kept the same shape for this function, (*s*,*k*) = (15.2 *years*, 1.9), but refitted the overall scale *A_max_* to the ART prevalence in HIV-infected individuals measured by the ZAMSTAR prevalence survey. Drop out from ART was taken as 5% per year. HIV-infected individuals with detected TB disease were initiated on ART with a specified coverage after 2010.

Individuals become non-infectious upon treatment initiation [18].

**Demography**

The model is populated in 1980 with 25,000 individuals whose characteristics are taken from the South African members of the ZAMSTAR prevalence survey. The results are multiplied up to the full population of SA at the end of each simulation. The initial prevalence of active TB was based on the WHO estimate of TB prevalence for 1990 and the number on treatment implied by the case-detection ratio. The appropriate numbers of active TB cases and individuals on TB treatment were selected randomly from amongst adults. The prevalence of latent *M.tb* infection was determined by assuming that the force-of-infection generated by this prevalence had been constant for long enough to achieve equilibrium. *M.tb* infections were distributed by age as for an equilibrium, as were the times since *M.tb* infection.

Age-specific mortality was implemented using the 1-parameter UN life-tables, with the calendar time dependence via the UN ESA estimates of life-expectancy of birth for each year [19], with the HIV-induced dip smoothed out. The UN ESA estimates of crude birth rate and in- and out-migration were also used, with immigrating individuals randomly cloning adults from the population. Finally, if necessary, a small correction was applied to birth and death rates if the relative population size over- or under-shot the UN ESA population growth prediction for South Africa, to ensure a matching trend.

**Model calibration**

The model was calibrated using a Nelder-Mead simplex algorithm to minimize a sum of squares error (SSE) term measuring the distance between TB, HIV and ART data and the estimates by WHO [10], UNAIDS [20], and ZAMSTAR [21]. The error term was constructed by rescaling each time-series to be comparable and additionally scaling the contribution at each time point by the inverse of the confidence interval. The total error was the sum of SSEs of this type for TB prevalence, TB incidence, HIV-TB incidence, TB incidence in 2010, HIV prevalence and ART coverage in all those with HIV infections in 2010. The parameters varied to achieve a fit were the effective contact rate, the remote reactivation rate, the probability of fast progression to pulmonary TB (PTB) for a naïve adult, the time to detection of TB in both HIV negatives and PLHIV, the protection from active disease if latently infected and the slope of the logarithm of the incidence rate ratio (IRR) against decline in CD4 count.

Table A: Model parameters and intervention parameter values used in the model. * values are fitted to data in the Basecase scenario.

| **Section** | | **Definition** | **Value in basecase** | **Value in intervention** | | **Notes** |
| --- | --- | --- | --- | --- | --- | --- |
| **TB in HIV-** | Contact rate | Effective contact rate (per year) | 9.58186* | / | |  |
|  | Survival | Weibull shape parameter for untreated TB survival | 1.5 | / | | ^22^ |
|  |  | Weibull scale parameter for untreated TB survival (years) | 2.25 | / | |  |
|  |  | Case-fatality rate for untreated TB | 0.551 | / | | Those on TB treatment are ~45% less likely to die than those not on treatment [22]. |
|  | Activation | Remote reactivation rate (per year) | 0.00333* | / | |  |
|  |  | Fast progression rate (per year) | 0.9 | / | | Assumption |
|  |  | Probability of fast progression to PTB for naïve adult | 0.13616* | / | |  |
|  | Case detection | Probability of detecting & starting treatment for TB | 0.69 | 0.771 | | The same as in PLHIV [23] |
|  |  | Odds ratio for detection & treatment: sputum smear - vs. sputum smear + | 0.7 | / | | Sputum smear negative TB is 30% less likely to be detected. |
|  |  | Detection timing as a proportion of time to death/self-cure | 0.36541* | / | |  |
|  | Treatment | Treatment success rate (clearance) | 0.79 | 0.895 | | Adherence is captured by only considering those who complete a full course of treatment as starting treatment [23]. |
|  |  | Treatment fatality rate if not successful | 0.3 | / | | Mortality rates on treatment for new TB cases in South Africa have remained around the 7% level from 2000 to 2009 [24]. If the treatment success rate is 77%, then the probability of death, if not someone who is successfully treated, is 7/(100-77) = 0.3. |
|  | Protection | Protection from progression to TB disease from latently infected | 0.64061* | / | |  |
|  |  | Relative infectiousness if sputum smear negative compared to sputum smear positive | 0.23 | / | | [7,8] |
| **HIV+ description** | Survival | Weibull shape parameter for untreated HIV survival | 2.3 | / | | Fitted to data from [13]. |
|  |  | Weibull scale parameter for untreated HIV survival (years) | 13.3 | / | | Fitted to data from [12]. |
|  | Infection | Weibull shape parameter for untreated HIV infection age | 2.3 | / | |  |
|  |  | Weibull scale parameter for untreated HIV infection age (years) | 25.9 | / | |  |
|  |  | CD4 count at HIV infection | 1000 | / | | An approximation similar to [14]. |
| **TB in HIV+** | Survival | Weibull shape parameter for untreated TB survival | 2 | / | | [15] |
|  |  | Weibull scale parameter for untreated TB survival (years) | 0.45 | / | |  |
|  | Sputum smear status | Relative risk of being sputum smear positive if HIV+ | 0.45 | / | | [25] |
|  |  | Slope of log(IRR) vs. CD4 decrement (10^4 L) | 0.61822* | / | |  |
|  | Case detection | Probability of detecting & starting treatment for TB | 0.69 | 0.771 | | The same as in HIV- [23] |
|  |  | Odds ratio for detection & treatment: sputum smear - vs. sputum smear + | 1 | / | |  |
|  |  | Detection timing as a proportion of time to death/self-cure | 0.36541* | / | |  |
|  |  | Treatment success rate (clearance) | 0.69 | 0.845 | | [23] |
|  |  | Treatment fatality rate if not success | 0.25 | / | |  |
|  | Link to care | CD4 increment above ART start when linked to care | 100 | / | | Some proportion of PLHIV who are set to receive ART, “learn” their status at an earlier time point. These can be targeted with pre-ART interventions. |
|  |  | Proportion starting ART who link to care earlier | 0.01 | / | |  |
| **ART** | Background | When ART use starts | 2005 | / | |  |
|  |  | Scale parameter for ART rollout (years) | 0.5 | / | | ART coverage reaches 70% by 2020 in those eligible. |
|  |  | Coverage parameter for ART rollout | 0.6 | / | |  |
|  | CD4 count | CD4 guideline for starting ART (<2012) | 200 | / | |  |
|  |  | CD4 count on ART | 750 | / | | Gives IRR ~0.3 |
|  | Impact | Influence of start-CD4 on life-expectancy on ART | 0.5 | / | | See model description above. |
|  | Timing | Shape parameter governing function for ART start timing | 7.2 | / | | See model description above. |
|  |  | Scale parameter governing function for ART start timing | 366.7 | / | |  |
|  |  | Rate at which those below CD4 guideline start ART. | 100 | / | | Catch-up mechanism for those not linked to care prior to ART. |
|  | Default | Default rate for PLHIV on ART (percentage per year) | 5 | / | | Unlike TB treatment, ART has a constant default rate. |
|  | Intervention | Coverage of ART initiation in HIV+ incident TB | 0.15 | / | |  |
|  | UTT | Period for testing rounds (years) | 1 | / | |  |
|  |  | Probability PLHIV are reached by a testing round and started on ART | / | 0.2/0.42 | | Coverage varies in different portfolios |
| **IPT** | Intervention | Coverage of IPT in PLHIV linking to care | 0.1 | 0.53 | | Coverage varies in different portfolios |
|  |  | Hazard ratio of progression to TB in HIV- | 0.37 | / | | A 63% protection whilst on IPT in line with individual-level analysis from Thibela [26] and [27].HIV does not impact this. |
|  |  | Hazard ratio of progression to TB in HIV+/ART- | 0.37 | / | |  |
|  |  | Hazard ratio of progression to TB in HIV+/ART+ | 0.5 | / | | The CD4 model here results in a 60% reduction in TB incidence at low CD4 counts. This is comparable to the Thibela assumptions (65%) [26]. The combined effect of ART and IPT in Thibela is ~80%. By having a hazard ratio of 0.5, at low CD4 the reduction will be 80%, decreasing with CD4 count to recreate the decreasing impact of ART at higher CD4. |
|  |  | Probability of clearance of TB at end of IPT course for HIV- | 0.1 | / | | A low level of clearance is predicted from Thibela, Botusa and recent modelling [28] and [29] |
|  |  | Probability of clearance of TB at end of IPT course for HIV+ | 0.1 | / | |  |
|  |  | Length of IPT treatment in for HIV- (years) | 0.5 | 3 / lifelong | | Length varies in different portfolios |
|  |  | Length of IPT treatment in for HIV+ (years) | 0.5 | 3 / lifelong | |  |
|  |  | Coverage of IPT intervention in HIV- | / | 0.2 | | 20% random coverage is considered. |
| **Periodic active case finding** | Intervention | Period between active case finding rounds (years) | 1 | Low | High |  |
|  |  | Probability of detection per round, sputum smear+ and HIV- | / | 0.24 | 0.32 | Screening offered to 60%, but only 80% uptake. 16% pre-treatment loss to follow-up. Sensitivity varies depending on algorithm (low/high). HIV status does not affect sensitivity. |
|  |  | Probability of detection per round, sputum smear- and HIV- | / | 0.16 | 0.24 |  |
|  |  | Probability of detection per round, sputum smear+ and HIV+ | / | 0.24 | 0.32 |  |
|  |  | Probability of detection per round, sputum smear- and HIV+ | / | 0.16 | 0.24 |  |
|  |  | Probability of detection per round, sputum smear+ and HIV+ and on ART | / | 0.24 | 0.32 |  |

**2) HIV incidence**

HIV incidence over time was generated using a dynamic transmission model built to investigate the PoPART intervention [30]. Each of the interventions thought to impact HIV incidence (change to ART guidelines) were simulated in the HIV model and the corresponding HIV incidence data included within the model (Figure B).

Figure B: HIV incidence over time for the Basecase and two interventions that affect HIV incidence.

**3) Details of individual interventions**

Interventions involve one of four control measures: ART, IPT, periodic active case finding or improved health care services.

**Basecase scenario**

Keeps all as in 2012, i.e. does not include the 80% coverage of ART to those co-infected with TB&HIV which is included in all Portfolios.

**1 Antiretroviral therapy (ART) guidelines**

PLHIV have an increased risk of active TB disease. This risk is decreased by the prescription of ART [31,32]. ART also decreases the probability of HIV transmission [33]. As TB risk and CD4 count are linked in this model, the impact of ART on TB incidence varies with time on ART as CD4 counts increase.

1. **ART to PLHIV and TB disease**

Current WHO guidelines recommend that all PLHIV with TB disease, irrespective of their CD4 count, be given ART in resource limited settings [34]. Within the NSP this is outlined as one of the strategies that will be immediately activated (Intervention 3.1.7 [35]). Despite relatively frequent screening of PLHIV for TB, prescription of ART is likely to be rare in line with IPT trends (see below). As such coverage was assumed to be 15% of PLHIV with TB in the current scenario. A higher coverage level of 80% was also investigated.

1. **CD4 guidelines for ART initiation change to 500 cells/mm^3^**

Current South African (as of August 2011[36]) and WHO guidelines recommend ART for PLHIV with CD4 counts <350 cells/mm^3^ [34]. Future strategies may increase this CD4 level, e.g. to <500 cells/mm^3^, or recommend universal ART use [37]. This intervention is not specifically outlined in the NSP but is implicit in their desire for increased control of the joint HIV/TB epidemics.

In the model, this is captured by a change to the guideline CD4 threshold for ART start (which affects only those linked to care) and a linear increase in the median CD4 count at which other PLHIV start ART to 500 cells/mm^3^. The shape of the distribution of starting CD4 counts was assumed to remain as it was in the Baseline scenario [38]. ART coverage was set to that reported in UNAIDS, for those with a CD4 count < 350 cells/mm^3^, of 80% by 2012. In the intervention, coverage of ART to those with a CD4 count < 500 cells/mm^3^ is assumed to already be 70% by 2014, rising to 90% by 2018.

**d) Universal test and treat (UTT)**

Testing high proportions of the South African population for HIV and initiating all PLHIV onto ART would help maintain the CD4 count of the population [34] but has issues such as long term antiretroviral (ARV) preservation.

Part of this intervention is included within the NSP with the aim to test all South Africans for HIV annually (Intervention 3.1.1 [35]). Current testing levels, imply that approximately 40% of the population were tested for HIV in 2009 [39]. Existing guidelines to place all PLHIV onto IPT claim to have achieved 53% coverage (as part of the HIV counselling and testing campaign 2010 [40]). Hence coverage of new testing and subsequent initiation of ART were estimated, from this limited data, at a baseline of 20%. The NSP aims for much higher testing levels (~80%), so a higher coverage of testing and ART initiation of 42% was also considered. UTT is assumed to take place annually. No additional case finding of TB was included in this HIV/ART only UTT intervention.

**2. Isoniazid preventive therapy (IPT)**

Isoniazid is a bactericidal antibiotic that is used in the first line combination treatment of tuberculosis. It is also prescribed to those at risk of TB disease, e.g. PLHIV, and has been shown to prevent them from becoming an active TB case [41,42]. However, the high protective effect is only whilst taking IPT; there appears to be little long-term protective effect of IPT once completed in high prevalence settings such as South Africa [26-28]. For all IPT scenarios, only a single course of IPT could be received in a lifetime.

1. **Isoniazid preventive therapy to all PLHIV for 6 months**
2. **36 months or**
3. **continuous**

South African guidelines currently outline that IPT should be give to PLHIV for different lengths of time dependent on their ART and TST status [43]. The minimum length of time was for 6months. TST status was not included in the model. Instead all PLHIV were eligible for IPT. Also investigated here, as suggested by D. Mametja at the 3^rd^ RSA TB conference and a current recommendation of the WHO [44], is 36 months of IPT, as well as continuous or lifelong IPT. The latter is under consideration by the WHO, but there is no trial evidence to support a change to this recommendation. However, with uncertainty as to the clearance efficacy of IPT but strong evidence for a large protective effect whilst taking IPT, lifelong IPT is likely to be effective at decreasing TB levels. Concerns with this length relate to resistance, however development was low after 36months of IPT [28].

Here, the probability of clearance, even at the end of 36 month therapy, is set to be 10% ^43^. Current estimates set there to be a 63% reduction in the probability of TB disease for those currently taking IPT, through reactivation or following reinfection, for individuals who are HIV negative or PLHIV who are not on ARVs [26,32,45]. For those PLHIV on ARVs, the effect appears to be multiplicative, with an 80% protective effect assumed [26,45].

IPT guidelines are poorly adhered to - only 38% of those PLHIV screened and found to be negative for TB are quoted as receiving IPT by the South Africa government [46]. Coverage was higher for PLHIV newly enrolled in HIV care (53% in a recent campaign), however the NSP target is 85% of those newly enrolled. WHO data suggests the coverage may be even lower at ~2%: 124,049 PLHIV were provided with IPT in 2010 [47] out of an estimated 5.63 million PLHIV [48]. Here, for individual interventions, low baseline coverage of IPT to PLHIV was estimated at 10%. A higher coverage, of 53%, was also considered.

1. **IPT to HIV negatives**

IPT can also be prescribed to those without HIV, currently often to those found via TB case contact tracing (e.g. [49]). Random allocation of IPT to the HIV negative community is a simple use of current technologies with the potential for widespread benefits [50], but must be balanced against the risk of side effects and resistance generation. Here 20% coverage of IPT for 6 months to those without HIV is investigated.

1. **IPT to PLHIV prior to ART**

IPT and ART have a similar beneficial preventative impact on TB disease individually whilst being taken, but in combination the impact is thought to be multiplicative (as assumed above). However, some studies have shown the combined impact to be not much greater than the individual impact [28] suggesting a lack of cost-effectiveness of co-prescription. There is also increased toxicity from co-prescription, especially when ART includes stavudine [51]. This has led Malawi, which has on going use of stavudine, to have HIV treatment guidelines recommending IPT only for PLHIV prior to ART start [52]. Here, the impact of such a policy with the same coverage as above (10%) in those linked to care prior to ART was investigated.

**3. Periodic active case finding for TB**

This intervention is outlined in the NSP, with an ambitious target of screening 30 million people annually for active TB (approximately 60% of the population) by 2016 (Intervention 3.1.1, [35]). Repeated, frequent screening for TB should reduce the number of undetected cases and reduce transmission rates. Such an intensive intervention such as this will be expensive, but this must be weighed against the cost of the on going HIV/TB epidemic in South Africa, which needs tackling through bigger and better interventions [53]. This is taken to be different to the strategies employed in ZAMSTAR – here this is assumed to be an active round of screening (door to door for example) and not just better availability of testing and community mobilisation as in ZAMSTAR [54].

Here, for simplicity, annual screening is assumed to take place on January 1^st^ with varying probabilities of detection linked to sputum smear and HIV status. This type of annual screening is assumed to be via a mobile active screening unit such as used in a recent study in South Africa [55] and such as was shown to be most effective in an earlier comparative study of active case finding methods [56].

1. **Low and**
2. **High sensitivity algorithm**

Here, the range of possible diagnostic algorithms and their sensitivities is simplified to two scenarios. The first aims to capture a low cost, low sensitivity algorithm, likely to involve smear microscopy. Sputum smear microscopy is valuable as sputum smear positive people are the most transmissible and therefore the most important target for screening and treatment. The second scenario captures a more effective but possibly a more expensive algorithm, for example based on GeneXpert. The use of culture would increase the case detection rate but would have problems with reporting results back to patients [55] and longer delays. As South Africa has achieved a rapid scale-up of GeneXpert coverage, here use of the low sensitivity algorithm is only considered in the supplementary.

Assuming that this is one of the main interventions the South Africa government will pursue, coverage of periodic active case finding is taken to be either the 60% aimed for or 30%, as achieved in recent HIV testing campaigns [24]. However, not all those reached will agree to testing – approximately 80% of people contacted through active case finding undergo screening [55,56]. Hence coverage of screening was set at 48%. The two screening algorithms then have different effectiveness dependent on sputum status. For the high scenario these are 80% and 60% for sputum smear positive and negative respectively. For the low scenario, 60% and 40%. HIV status is assumed to not impact sensitivity [57]. Detection of TB in PLHIV on ART is assumed to be the same as in someone without HIV. The percentage of patients lost to follow-up (‘primary default’) [58] is included at 16% [55]. The values for high coverage and low and high case detection are then 24%/16%^[[1]](#footnote-1)^ and 32%/24% for detection and treatment initiation of sputum smear positive/negative pulmonary TB respectively. For low coverage the numbers, with high case detection, would be 16%/12% for sputum smear positive/negative pulmonary TB respectively.

**4. 50% decrease in loss of patients to follow-up (‘primary default’)**

Within South Africa, there is a large gap between the number of patients being screened for TB via symptom screening and/or microbiologically and the number who are positive who then return for treatment. Around 17-21% of those found to have active TB are lost to follow-up before or during a course of TB treatment [59-61]. This intervention would be implemented by improved, more pro-active health services with more effective follow-up procedures and the use of quicker diagnostics.

The proportion of patients detected and started on treatment is approximated using the data from the WHO for case detection rates in new and relapse cases (69%) [62]. Halving the loss to follow-up level (19%) increases the percentage starting treatment to 77%. PLHIV have the same case detection rates but a different proportion of infections that become active.

**5. 50% increase in treatment success**

Treatment success could be simply improved by better adherence and improved health service systems – both via better education and via increased coverage of directly observed treatment, short-course (DOTS). Several new drugs and regimens for TB are also currently under development [63]. These have the potential to dramatically decrease treatment time and may have the potential to out perform current drugs. The impact of such improvements or a new regimen is here captured by decreasing by 50% the proportion of patients that fail at the end of treatment.

Here, treatment success depends on HIV status. The percentage of sputum smear positive TB patients that adhered to treatment and were cured in South Africa in 2010 was 79% [62]. This is taken as the baseline treatment success level for those without HIV. Adherence to treatment in PLHIV is lower than in those without HIV by approximately 10% [64]. However, treatment failure is approximately the same at 2-4% [64-66]. Treatment success is then set at 69% for PLHIV. Increased treatment success raises this percentage to 85% and 90% for PLHIV and without HIV respectively.

**4) Results of the impact of individual interventions**

The impact of all the individual interventions and the portfolios on TB disease incidence and TB deaths relative to the 2012 values are shown in Table B. The coverage levels investigated in different portfolios are included as are the varying sensitivities for the active case finding algorithms. Several interventions are included which are not included in the portfolios, such as IPT pre-ART.

As shown in Figure B for the NSP portfolio, the contributions of the various interventions to each of the other portfolios is shown in Figures C-E.

Table B: Reduction in TB incidence and TB deaths by each portfolio. Values in bold and underlined indicate where the reductions are greater than the targets at this time point (for 2016 and 2032).

|  | | | **2016** | | **2032** | | **2050** | |
| --- | --- | --- | --- | --- | --- | --- | --- | --- |
|  | **NSP target** | | 50% reduction | | Zero new cases or deaths | |  | |
|  | **Control method (coverage)** | | **Incidence** | **Mortality** | **Incidence** | **Mortality** | **Incidence** | **Mortality** |
| **Portfolio** | **Basecase** | | 13 | 15 | 18 | 26 | 14 | 21 |
|  | **NSP** | | 32 | **57** | 55 | 75 | 61 | 80 |
|  | **WHO** | | 22 | 21 | 32 | 40 | 22 | 31 |
|  | **Novel strategies** | | 32 | 39 | 29 | 40 | 20 | 29 |
|  | **Optimised** | | 46 | **71** | 70 | 86 | 76 | 89 |
| **ART** | **ART to PLHIV with TB** | **(15%)** | 10 | 13 | 14 | 21 | 11 | 18 |
|  |  | **(80%)** | 10 | 14 | 11 | 19 | 8 | 15 |
|  | **CD4 guideline change to 500** |  | 10 | 13 | 21 | 30 | 17 | 26 |
|  | **Universal test and treat** | **(20%)** | 19 | 24 | 21 | 30 | 16 | 24 |
|  |  | **(42%)** | 25 | 32 | 23 | 33 | 18 | 27 |
| **IPT to PLHIV** | **For 6months** | **(10%)** | 9 | 12 | 14 | 21 | 12 | 19 |
|  |  | **(53%)** | 10 | 13 | 14 | 22 | 12 | 19 |
|  | **For 36months** | **(10%)** | 9 | 12 | 14 | 21 | 12 | 19 |
|  |  | **(53%)** | 10 | 13 | 15 | 22 | 12 | 19 |
|  | **Continuous (10%)** | | 9 | 12 | 15 | 22 | 12 | 19 |
|  | **IPT pre-ART (10%)** | | 9 | 12 | 14 | 21 | 12 | 19 |
| **IPT to HIV-** | **For 6months (20%)** | | 16 | 17 | 17 | 25 | 13 | 20 |
| **Periodic active case finding** | **Low sensitivity, high coverage** | | 20 | 28 | 42 | 52 | 49 | 58 |
|  | **High sensitivity, low coverage** | | 16 | 23 | 32 | 42 | 35 | 45 |
|  | **High sensitivity, high coverage** | | 24 | 33 | 48 | 58 | 58 | 67 |
| **Loss to follow up** | **50% decrease** | | 12 | 35 | 30 | 52 | 33 | 54 |
| **Treatment success** | **50% increase** | | 13 | 22 | 17 | 31 | 15 | 28 |

**Figure C: Impact of “WHO” portfolio and component interventions on TB incidence**

**Figure D: Impact of “Novel strategies” portfolio and component interventions on TB incidence**

**Figure E: Impact of “Optimized” portfolio and component interventions on TB incidence.**

**5) Sensitivity analysis**

A full uncertainty analysis could not be performed for this model as it would be too computationally expensive. Instead we investigated the impact of altering a set of key model parameters (Table C) on the key outcome – would the “NSP” portfolio ever achieve the 2016 and 2032 NSP targets? This set of parameters was chosen from the subset of parameters that are not used to generate the TB model calibration and that were linked to key interventions within the “NSP” portfolio.

Table C: Key model parameters varied in sensitivity analysis.

| **Parameter** | **Value in main analysis** | **Value explored in sensitivity analysis** | | **Reason for choice** |
| --- | --- | --- | --- | --- |
|  |  | **Low** | **High** |  |
| *tbart* (probability that TB on ART is like HIV-) | 1 | 0.5 | 0.8 | NSP includes high coverage of ART to those co-infected with TB. This impact would be moderated if ART had less of an impact on TB disease, making it more like TB disease in PLHIV. |
| *Iprotn*  (protection from infection if latent) | 0 | 0.2 | 0.5 | NSP includes IPT to PLHIV. This impact will be moderated if there is already protection from latency. |
| *Rxpd*  (Probability of death if not cleared) | 0.3 | 0.15 | 0.45 | Variation to investigate healthcare interventions. |
| *deltansmr*  (Ratio of CDR in smr-/+) | 0.7 | 0.5 | 0.9 |  |

***Results***

One-way sensitivity analysis shows that varying a set of natural history parameters has little influence on the impact of the “NSP” portfolio (Table D, Figure F). The only parameter which generated a greater than 3% difference was *tbart* suggesting that a driver of the impact of the portfolios is the effect of ART on the risk of acquiring TB in those with HIV. Lowering the parameter, decreases the impact of the NSP (negative values in Figure F) reflecting that the ART components of NSP will have a greater impact if ART makes those with HIV exactly like those without HIV (baseline value of *tbart* ==1). The other parameters have little effect on the impact.

Table D: Reduction in TB incidence and mortality, with different parameter values, from 2012 levels. “Y” indicates where the reductions are greater than the targets at this time point. An “X” indicates where the reduction is less than the NSP target for 2016 or 2032 or not at the 2032 target by 2050.

|  | |  | **2016** | | **2032** | | **2050** | |
| --- | --- | --- | --- | --- | --- | --- | --- | --- |
| **NSP target** | |  | 50% reduction | | Zero new cases and deaths | |  | |
|  | |  | **Incidence** | **Mortality** | **Incidence** | **Mortality** | **Incidence** | **Mortality** |
|  | *Baseline* |  | X | Y | X | X | X | X |
| **Natural history parameters** | *tbart* | low | X | Y | X | X | X | X |
|  |  | high | X | Y | X | X | X | X |
|  | *Iprotn* | low | X | Y | X | X | X | X |
|  |  | high | X | Y | X | X | X | X |
|  | *Rxpd* | low | X | Y | X | X | X | X |
|  |  | high | X | Y | X | X | X | X |
|  | *deltansmr* | low | X | Y | X | X | X | X |
|  |  | high | X | Y | X | X | X | X |

Figure F: Different in percentage reduction in TB incidence from 2012 value by 2050 for one-way sensitivity analysis of four model parameters. In blue is the output for low parameter values, red for high values. Plotted here is the percentage reduction with the changed parameters at 2050 minus the percentage reduction at 2050 in the NSP portfolio from the main analysis.

References

1. Dodd PJ, White RG, Corbett EL (2011) Periodic active case finding for TB: when to look? PLoS One 6: e29130.

2. White RG, Glynn JR, Orroth KK, Freeman EE, Bakker R, et al. (2008) Male circumcision for HIV prevention in sub-Saharan Africa: who, what and when? AIDS 22: 1841-1850.

3. Consortium to Respond Effectively to the TB/AIDS Epidemic.

4. Dodd PJ, Looker C, Plumb I, Bond G, Schaap A, et al. (In review) *Mycobacterium tuberculosis infection* incidence and social contact patterns in Zambia and South Africa. In: Unpublished, editor.

5. Vynnycky E, Fine PE (1997) The annual risk of infection with Mycobacterium tuberculosis in England and Wales since 1901. Int J Tuberc Lung Dis 1: 389-396.

6. Andrews JR, Noubary F, Walensky RP, Cerda R, Losina E, et al. (2012) Risk of progression to active tuberculosis following reinfection with *Mycobacterium tuberculosis*. Clin Infect Dis 54: 784-791.

7. Behr MA, Warren SA, Salamon H, Hopewell PC, Ponce de Leon A, et al. (1999) Transmission of *Mycobacterium tuberculosis* from patients smear-negative for acid-fast bacilli. Lancet 353: 444-449.

8. Tostmann A, Kik SV, Kalisvaart NA, Sebek MM, Verver S, et al. (2008) Tuberculosis transmission by patients with smear-negative pulmonary tuberculosis in a large cohort in the Netherlands. Clin Infect Dis 47: 1135-1142.

9. Tiemersma EW, van der Werf MJ, Borgdorff MW, Williams BG, Nagelkerke NJ (2011) Natural history of tuberculosis: duration and fatality of untreated pulmonary tuberculosis in HIV negative patients: a systematic review. PLoS One 6: e17601.

10. WHO (2013) TB data. Available: http://www.who.int/tb/country/en/. Accessed 2015 March 2

11. Fraser C, Cori A (2012) HIV model to investigate the PopART intervention.

12. Stover J, Johnson P, Hallett T, Marston M, Becquet R, et al. (2010) The Spectrum projection package: improvements in estimating incidence by age and sex, mother-to-child transmission, HIV progression in children and double orphans. Sex Transm Infect 86 Suppl 2: ii16-21.

13. Collaborative Group on AIDS Incubation and HIV Survival including the CASCADE EU Concerted Action C (2000) Time from HIV-1 seroconversion to AIDS and death before widespread use of highly-active antiretroviral therapy: a collaborative re-analysis. . Lancet 355: 1131-1137.

14. Williams BG, Granich R, De Cock KM, Glaziou P, Sharma A, et al. (2010) Antiretroviral therapy for tuberculosis control in nine African countries. Proc Natl Acad Sci U S A 107: 19485-19489.

15. Dimairo M, Mativenga S, Dauya E, Makamure B, Mangwanya D, et al. (2009) The fate of sputum smear-negative TB suspects managed by routine clinical services in Harare, Zimbabwe. CROI. Montreal, Canada.

16. Dodd PJ, Knight GM, Lawn SD, Corbett EL, White RG (2013) Predicting the long-term impact of antiretroviral therapy scale-up on population incidence of tuberculosis. PLoS One 8: e75466.

17. Eaton JW, Menzies NA, Stover J, Cambiano V, Chindelevitch L, et al. (2013) Health benefits, costs, and cost-effectiveness of earlier eligibility for adult antiretroviral therapy and expanded treatment coverage: a combined analysis of 12 mathematical models. Lancet Global Health.

18. Sepkowitz KA (1996) How contagious is tuberculosis? Clin Infect Dis 23: 954-962.

19. Department of Economics and Social Affairs PD (2011) World Population Prospects: The 2010 Revision. In: United Nations, editor. New York.

20. UNAIDS (2013) AIDSinfo. Available: Accessed

21. Ayles HM, Sismanidis C, Beyers N, Hayes RJ, Godfrey-Faussett P (2008) ZAMSTAR, The Zambia South Africa TB and HIV Reduction Study: design of a 2 x 2 factorial community randomized trial. Trials 9: 63.

22. NTI (1981) A study of the characteristics and course of sputum smear-negative pulmonary tuberculosis. Tubercle 62: 155-167.

23. WHO (2013) Global Tuberculosis Control.

24. SANAC (2012) Global AIDS Response Progress Report, South Africa. Available: http://www.unaids.org/en/dataanalysis/knowyourresponse/countryprogressreports/2012countries/ce_ZA_Narrative_Report.pdf. Accessed 2015 March 2

25. Getahun H, Harrington M, O'Brien R, Nunn P (2007) Diagnosis of smear-negative pulmonary tuberculosis in people with HIV infection or AIDS in resource-constrained settings: informing urgent policy changes. Lancet 369: 2042-2049.

26. Churchyard GJ, Fielding KL, Lewis JJ, Coetzee L, Corbett EL, et al. (2014) A trial of mass isoniazid preventive therapy for tuberculosis control. N Engl J Med 370: 301-310.

27. Comstock GW (1962) Isoniazid prophylaxis in an undeveloped area. Am Rev Respir Dis 86: 810-822.

28. Samandari T, Agizew TB, Nyirenda S, Tedla Z, Sibanda T, et al. (2011) 6-month versus 36-month isoniazid preventive treatment for tuberculosis in adults with HIV infection in Botswana: a randomised, double-blind, placebo-controlled trial. Lancet 377: 1588-1598.

29. Vynnycky E, ... (Draft) Thibela TB didn’t do it. What might control TB in the mines in Southern Africa? . Unpublished.

30. Eaton JW, Johnson LF, Salomon JA, Barnighausen T, Bendavid E, et al. (2012) HIV Treatment as Prevention: Systematic Comparison of Mathematical Models of the Potential Impact of Antiretroviral Therapy on HIV Incidence in South Africa. PLoS Med 9.

31. Badri M, Wilson D, Wood R (2002) Effect of highly active antiretroviral therapy on incidence of tuberculosis in South Africa: a cohort study. Lancet 359: 2059-2064.

32. Suthar AB, Lawn SD, Del Amo J, Getahun H, Dye C, et al. (2012) Antiretroviral Therapy for Prevention of Tuberculosis in Adults with HIV: A Systematic Review and Meta-Analysis. PLoS Med 9: e1001270.

33. Cohen MS, Chen YQ, McCauley M, Gamble T, Hosseinipour MC, et al. (2011) Prevention of HIV-1 infection with early antiretroviral therapy. N Engl J Med 365: 493-505.

34. WHO (2010) Antiretroviral therapy for HIV infection in adults and adolescents. Recommendations for a public health approach. 2010 revision. .

35. South African National Aids Council (SANAC) (2012) National Strategic Plan on HIV, STIs and TB 2012-2016.

36. SANAC (2011) Statement on the meeting of the South African National AIDS Council (SANAC).

37. Cohen MS, Muessig KE, Smith MK, Powers KA, Kashuba AD (2012) Antiviral agents and HIV prevention: controversies, conflicts, and consensus. AIDS 26: 1585-1598.

38. Nash D, Wu Y, Elul B, Hoos D, El Sadr W (2011) Program-level and contextual-level determinants of low-median CD4+ cell count in cohorts of persons initiating ART in eight sub-Saharan African countries. AIDS 25: 1523-1533.

39. Johnson S, Kincaid L, Laurence S, Chickwava F, Delate R, et al. (2010) Second National HIV Comunication Survey 2009. Pretoria: Johns Hopkins Health and Education in South Africa.

40. SANAC (2010) The National HIV counselling and testing campaign and strategy.

41. Akolo C, Adetifa I, Shepperd S, Volmink J (2010) Treatment of latent tuberculosis infection in HIV infected persons. Cochrane Database Syst Rev: CD000171.

42. Smieja MJ, Marchetti CA, Cook DJ, Smaill FM (2000) Isoniazid for preventing tuberculosis in non-HIV infected persons. Cochrane Database Syst Rev: CD001363.

43. Department of Health RoSA (2013) The South African Antiretroviral Treatment Guidelines. In: Health Do, editor.

44. WHO (2011) Guidelines for intensified tuberculosis case-finding and isoniazid preventive therapy for people living with HIV in resource-constrained settings. World Health Organization, Geneva, Switzerland: Department of HIV/AIDS and Stop TB Department.

45. Golub JE, Saraceni V, Cavalcante SC, Pacheco AG, Moulton LH, et al. (2007) The impact of antiretroviral therapy and isoniazid preventive therapy on tuberculosis incidence in HIV-infected patients in Rio de Janeiro, Brazil. AIDS 21: 1441-1448.

46. NDOH (2011) Programme Data.

47. WHO (2011) Global Tuberculosis Control.

48. Africa ASoS (2011).

49. Pothukuchi M, Nagaraja SB, Kelamane S, Satyanarayana S, Shashidhar, et al. (2011) Tuberculosis contact screening and isoniazid preventive therapy in a South Indian district: operational issues for programmatic consideration. PLoS One 6: e22500.

50. Zelner JL, Murray MB, Becerra MC, Galea J, Lecca L, et al. (2014) Bacillus Calmette-Guérin and isoniazid preventive therapy protect contacts of tuberculosis patients. Am J Respir Crit Care Med.

51. Breen RA, Lipman MC, Johnson MA (2000) Increased incidence of peripheral neuropathy with co-administration of stavudine and isoniazid in HIV-infected individuals. AIDS 14: 615.

52. Ministry of Health M (2011) Clinical Management of HIV in Children and Adults. Malawi.

53. Abdool Karim SS, Churchyard GJ, Abdool Karim Q, Lawn SD (2009) HIV infection and tuberculosis in South Africa: an urgent need to escalate the public health response. Lancet 374: 921-933.

54. Ayles H, Muyoyeta M, Du Toit E, Schaap A, Floyd S, et al. (2013) Effect of household and community interventions on the burden of tuberculosis in southern Africa: the ZAMSTAR community-randomised trial. Lancet 382: 1183-1194.

55. Kranzer K, Lawn SD, Meyer-Rath G, Vassall A, Raditlhalo E, et al. (2012) Feasibility, Yield, and Cost of Active Tuberculosis Case Finding Linked to a Mobile HIV Service in Cape Town, South Africa: A Cross-sectional Study. PLoS Med 9: e1001281.

56. Corbett EL, Bandason T, Duong T, Dauya E, Makamure B, et al. (2010) Comparison of two active case-finding strategies for community-based diagnosis of symptomatic smear-positive tuberculosis and control of infectious tuberculosis in Harare, Zimbabwe (DETECTB): a cluster-randomised trial. Lancet 376: 1244-1253.

57. Lewis JJ, Charalambous S, Day JH, Fielding KL, Grant AD, et al. (2009) HIV infection does not affect active case finding of tuberculosis in South African gold miners. Am J Respir Crit Care Med 180: 1271-1278.

58. Zachariah R, Harries AD, Srinath S, Ram S, Viney K, et al. (2012) Language in tuberculosis services: can we change to patient-centred terminology and stop the paradigm of blaming the patients? Int J Tuberc Lung Dis 16: 714-717.

59. Botha E, den Boon S, Lawrence KA, Reuter H, Verver S, et al. (2008) From suspect to patient: tuberculosis diagnosis and treatment initiation in health facilities in South Africa. Int J Tuberc Lung Dis 12: 936-941.

60. Botha E, Den Boon S, Verver S, Dunbar R, Lawrence KA, et al. (2008) Initial default from tuberculosis treatment: how often does it happen and what are the reasons? Int J Tuberc Lung Dis 12: 820-823.

61. Dunbar R, Lawrence K, Verver S, Enarson DA, Lombard C, et al. (2011) Accuracy and completeness of recording of confirmed tuberculosis in two South African communities. Int J Tuberc Lung Dis 15: 337-343.

62. WHO (2012) Global Tuberculosis Control.

63. Stop TB Partnership (2012) Working Group on New TB Drugs.

64. Kassim S, Sassan-Morokro M, Ackah A, Abouya LY, Digbeu H, et al. (1995) Two-year follow-up of persons with HIV-1- and HIV-2-associated pulmonary tuberculosis treated with short-course chemotherapy in West Africa. AIDS 9: 1185-1191.

65. Perriëns JH, St Louis ME, Mukadi YB, Brown C, Prignot J, et al. (1995) Pulmonary tuberculosis in HIV-infected patients in Zaire. A controlled trial of treatment for either 6 or 12 months. N Engl J Med 332: 779-784.

66. el-Sadr WM, Perlman DC, Matts JP, Nelson ET, Cohn DL, et al. (1998) Evaluation of an intensive intermittent-induction regimen and duration of short-course treatment for human immunodeficiency virus-related pulmonary tuberculosis. Terry Beirn Community Programs for Clinical Research on AIDS (CPCRA) and the AIDS Clinical Trials Group (ACTG). Clin Infect Dis 26: 1148-1158.

1. 80% reached, 60% screened, 60% effectiveness, (1-16) start treatment. Thus 0.8*0.6*0.6*0.84 = 0.2419 = 24%. With 40% effectiveness for smear negatives this reduces to 16%. [↑](#footnote-ref-1)
